# Increased Otolin-1 in Serum as a Potential Biomarker for Idiopathic Benign Paroxysmal Positional Vertigo Episodes

**DOI:** 10.3389/fneur.2020.00367

**Published:** 2020-05-13

**Authors:** Yunqin Wu, Weiwei Han, Wang Yan, Xiaoxiong Lu, Min Zhou, Li Li, Qiongfeng Guan, Zhenyi Fan

**Affiliations:** ^1^Department of Neurology, Hwa Mei Hospital, University of Chinese Academy of Science, Ningbo, China; ^2^Ningbo Institute of Life and Health Industry, University of Chinese Academy of Science, Ningbo, China; ^3^Department of Rehabilitation, Hwa Mei Hospital, University of Chinese Academy of Science, Ningbo, China

**Keywords:** otoconia, otolin-1, biomarker, vertigo, benign paroxysmal positional vertigo

## Abstract

**Objective:** Otolin-1, a main specific otoconia matrix protein, passes through the labyrinth-blood barrier and is detectable in peripheral blood. Serum otolin-1 levels differ between patients with benign paroxysmal positional vertigo (BPPV) and healthy controls and are significantly age-related, increasing in healthy controls with age, suggesting that serum otolin-1 levels reflect otolith status. The aim of this study was to determine whether otolin-1 levels change during vertigo episodes in patients with BPPV and whether any change is specific and sensitive enough for BPPV episodes.

**Method:** Patients diagnosed with *de novo* idiopathic BPPV during an acute episode were included in the study from May 2017 to May 2018. Blood samples were drawn before patients were treated with canalith-repositioning maneuvers. Serum otolin-1 levels were compared between 78 patients and 121 age- and sex-matched healthy individuals.

**Results:** There were no significant differences between the groups in the age distribution, sex ratio, body mass index, clinical history, routine blood parameters, or total protein, albumin, uric acid, creatinine, blood urea nitrogen and lipid profiles (*P* > 0.05). Serum levels of otolin-1 were significantly higher in BPPV patients than in healthy controls (*P* < 0.001). Receiver operating characteristic analysis revealed that a serum otolin-1 value of 299.45 pg/ml was the optimal cut-off value to discriminate patients with BPPV from healthy controls (area under the curve 0.757, 95% CI 0.687~0.826) with a sensitivity of 67.9% and a specificity of 72.7%.

**Conclusion:** Serum levels of otolin-1 may be a potential biomarker for BPPV episodes.

## Introduction

Benign paroxysmal positional vertigo (BPPV) is one of the most common otoconia-related balance disorders, accounting for 36.5% of all dizziness complaints in the Chinese population (1, 2). BPPV is characterized by transient vertigo, nausea and nystagmus provoked by head position changes. BPPV diagnosis mainly relies on a typical history and positive provocative maneuvers (1, 3). However, the diagnosis and management of nearly 30% of BPPV cases remain challenging, especially in subjective BPPV or cases with multiple canal involvement, and performing diagnostic positional maneuvers (e.g., in small children or frail elderly patients) can be difficult (4, 5). According to clinical practice guidelines, canalith repositioning maneuvers are recommended as the first approach to treat BPPV. Observation or “watchful waiting” is also a therapeutic option for BPPV (1, 6). Thus, the unnecessary examination and test of BPPV may result in increased costs for patients. Considering the high incidence of BPPV, it places a heavy burden on health care systems and society. Given these properties of BPPV, it is principally important to clarify its diagnosis. However, no laboratory indicators are currently available for establishing the diagnosis of BPPV.

Currently, it is widely accepted that the pathogenesis of BPPV involves the displacement of otoconia, which float into the semicircular canals or attach to the cupula of the semicircular canals, making them sensitive to gravity (7). Otoconia, as bone, are composed of Ca carbonate arranged as calcite crystals and are predominantly composed of the glycoproteins otolin-1 and otoconin 90, which make an organic core synthesized primarily during embryonic development, with calcification essentially completed by the seventh postnatal day (8–11). Otoconia are a dynamic calcium reservoir, and their repair and regeneration may occur throughout life (12–14). Dislodged otoconia can be dissolved in the endolymph, and matrix proteins are likely to be reabsorbed from the endolymph and released into circulation; however, little is known about these processes (15, 16). If proteins corresponding to inner ear diseases can be identified and tested with non-invasive techniques, then they have the potential to serve as biomarkers of inner ear health.

Otolin-1 is a secreted glycoprotein present mainly in otoconial crystals and fibrous membranes (17). It serves as a scaffolding protein that connects otoliths and otoconial core matrix proteins to the inner ear sensory epithelial and acellular gel matrix. Recently, studies reported that otolin-1 can be detected in serum and that its levels significantly increase with age, consistent with the age-related degeneration of otoconia (18–20). A preliminary study found that only one-third of patients with BPPV had higher serum otolin-1 values than those found in healthy controls because some of the enrolled BPPV patients were not in the acute stage (21). This promising result suggests that otolin-1 might have the potential to serve as a biomarker for acute episodes of BPPV.

The aim of this study was, therefore, to explore whether serum levels of otolin-1 can serve as a biomarker for distinguishing between acute episodes of BPPV and matched healthy controls without vertigo symptoms.

## Methods

All consecutive patients with a final diagnosis of *de novo* idiopathic BPPV during the attacks of vertigo at the Department of Neurology and Emergency, Hwa Mei Hospital, University of Chinese Academy of Science between May 2017 and May 2018 were included in this study. The diagnosis of BPPV was based on a typical history of recurrent, brief positional vertigo and clinical observation of characterized nystagmus during provocative maneuvers. The details of the diagnosis of BPPV were obtained according to the criteria established by the Barany Society (1, 3).

Some patients with persistent untreatable dizziness underwent head imaging, and other examinations were performed to exclude central nervous system disease. Patients with any history of head trauma, migraine, vestibular neuritis, Meniere's disease, sudden hearing loss, otitis media, ear surgery, severe organ dysfunction (e.g., chronic renal failure or liver or bile duct disease), malignant tumor or hormonal disorders were excluded. In addition, patients previously diagnosed with BPPV or multiple canal involvement were also excluded. The healthy control group included 121 volunteers with age and gender distributions similar to those of the study group; these individuals without a history of vertigo or dizziness were selected from the health check-up center of our hospital. We recorded all of the following data: age, sex, lifestyle habits, ongoing health problems, medication history, affected semicircular canal, onset time, initial assessment time and laboratory indicators.

This study was approved by our institutional review board (protocol number KY-2017-014-03) and adhered to the tenets of the Declaration of Helsinki. Informed consent was obtained from all subjects or his or her legal representative.

### Measurement of Otolin-1 and Other Parameters

Morning fasting blood samples were collected from all subjects and centrifuged at 3000 rpm for 10 min at 4°C. Serum was then separated and frozen at −80°C until assays were performed.

Otolin-1 was measured using a human otolin-1 enzyme linked immunosorbent assay (ELISA) kit (QAYEEBIO, Shanghai, China) as described in the manufacturer's instruction manual, and each sample was tested in triplicate. Absorbance was measured using a microplate reader (Molecular Devices Spectra Max Plus 384) at a wavelength of 450 nm. Other parameters consisted of hematological and biochemical analyses were measured using an automated machine at the laboratory of our hospital. The laboratory staff who analyzed the samples were blinded to the group assignment of the study participants.

### Statistical Analysis

SPSS Statistics 22.0 (SPSS Inc., Chicago, IL, USA) was used to analyse all the data. The Kolmogorov-Smirnov test was used to test the data distribution. Quantitative values following normal distributions are expressed as the mean ± SD; if not, they are presented as the median and interquartile range (IQR). Qualitative variables are described as numbers and percentages. A *T*-test, chi-square test, Fisher's test or non-parametric Mann–Whitney *U*-test was used to compare the differences between groups. Receiver operating characteristic (ROC) analysis was performed to determine the sensitivity and specificity of serum otolin-1 for distinguishing patients with BPPV from healthy controls. All *P*-values < 0.05 were considered statistically significant.

## Result

### Demographics and Clinical Characteristics of the Subjects

A total of 78 patients diagnosed with *de novo* idiopathic BPPV at our institution were included during the study period. After excluding 14 patients who were diagnosed with secondary BPPV, 10 patients who were previously diagnosed with BPPV, 4 patients who refused to participate in the study, 3 patients with severe organ dysfunction, and 1 patient in whom both the posterior and horizontal canals were affected, the final study group comprised 52 women and 26 men, resulting in a female to male ratio of 2:1. The patient ages ranged from 33 to 81 years (mean age 62.7 ± 10.7), and there was a significant difference between women and men (60.7 ± 12.0 vs. 66.7 ± 5.9, *p* = 0.004). In addition, the prevalences of smoking and drinking were significantly higher in men than in women (Table 1). However, there were no significant differences in the age distribution, sex ratio, body mass index, clinical history, lipid profiles, blood panels, or blood urea nitrogen levels between the BPPV patients and healthy controls (Table 2).

**Table 1:** Clinical characteristics of benign paroxysmal positional vertigo patients.

	**Female (52)**	**Male (26)**	***P***
Age (years)	60.7 ± 12.0	66.7 ± 5.9	0.004
BMI (kg/m^2^)	23.68 ± 3.23	23.10 ± 3.23	0.462
Hypertension [*n*(%)]	22 (42.3%)	12 (46.153%)	0.747
Diabetes [*n*(%)]	8 (15.38%)	5 (19.23%)	0.667
Smoking[*n*(%)]	4 (7.69%)	11 (42.307%)	0.001^*^
Drinking [*n*(%)]	5 (9.615%)	9 (34.615%)	0.007
Symptom onset to initial evaluation (D)	3.00 (1.00–5.75)	5.00 (1.00–7.00)	0.419
Symptom onset to blood sampling (D)	3.52 (1.66-6.06)	5.30 (1.76–7.50)	0.504
Subtype of BPPV			
PSCC [*n*(%)]	34 (65.4%)	15 (57.7%)	
LSCC canalolithiasis[*n*(%)]	13 (25.0%)	7 (26.9%)	
LSCC cupulolithiasis[*n*(%)]	5 (9.6%)	4 (15.4%)	

Values are expressed as n (%), mean ± SD or median (interquartile range). P-values were calculated using an independent t-test, chi-squared test, Fisher's test or non-parametric Mann–Whitney U-test. PSCC, posterior semicircular canal; LSCC, lateral semicircular canal. P-values < 0.05 were considered significant.

* *Fisher's test*.

**Table 2:** Demographic and biochemical characteristics of benign paroxysmal positional vertigo patients and healthy controls.

	**BPPV (*n* = 78)**	**Control (*n* = 121)**	***P***
Age (years)	62.7 ± 10.7	61.4 ± 11.9	0.434
Sex (M/F)	52/26	79/42	0.841
BMI (kg/m^2^)	23.48 ± 3.22	23.76 ± 2.98	0.534
Hypertension [*n*(%)]	34 (43.589%)	63 (52.06%)	0.243
Diabetes [*n*(%)]	13 (16.667%)	24 (19.83%)	0.575
Smoking[*n*(%)]	15 (19.23%)	31 (25.61%)	0.297
Drinking [*n*(%)]	14 (17.948%)	28 (23.14)	0.381
White blood cells (10^9^/L)	6.08 ± 1.85	6.04 ± 1.86	0.853
Hemoglobin (g/L)	129.4 ± 16.2	132.0 ± 15.7	0.252
Platelets (10^9^/L)	203.1 ± 45.6	191.7 ± 47.7	0.096
Total protein (g/L)	68.5 ± 5.9	68.0 ± 6.6	0.601
Albumin (g/L)	42.0 ± 3.7	41.8 ± 4.0	0.699
Creatinine (umol/L)	62.82 ± 15.57	66.09 ± 24.5	0.294
Blood urea nitrogen (mmol/L)	5.02 ± 1.29	5.25 ± 2.15	0.406
Uric acid (mmol/L)	293.68 ± 67.59	314.92 ± 107.79	0.122
Total cholesterol (mmol/L)	4.61 ± 1.27	4.60 ± 1.17	0.952
HDL-C (mmol/L)	1.28 ± 0.32	1.30 ± 0.33	0.712
LDL-C (mmol/L)	2.56 ± 0.91	2.58 ± 0.96	0.872
Triglycerides (mmol/L)	1.65 ± 1.61	1.57 ± 1.02	0.682

*BMI, body mass index was defined as weight in kilograms divided by the square of height in meters; HDL-C, high-density lipoprotein cholesterol; LDL-C, low-density lipoprotein cholesterol. Values are expressed as n (%) or mean ± SD. P-values were calculated using an independent t-test or the chi-squared test. P-values < 0.05 were considered significant*.

Of the 78 patients with BPPV, the most common finding was otoconia dislocated in the posterior canal (*n* = 49, 62.8%), followed by horizontal otoconia (*n* = 29, 37.2%), which included 20 cases of canalolithiasis and 9 of cupulolithiasis. The interval between the onset of symptoms and the initial evaluation varied from 6 h to 20 days (mean = 4.85 days, median = 3.00 days), and 79.5% of the patients were evaluated within a week from symptom onset. The time between blood collection and symptom onset varied from 18 h to 20.5 days (mean = 5.38 days, median = 3.65 days), and 74.4% of the blood samples were collected within 7 days of symptom onset.

### Serum Otolin-1 Levels in Patients With BPPV and Healthy Controls

**Figure 1:**
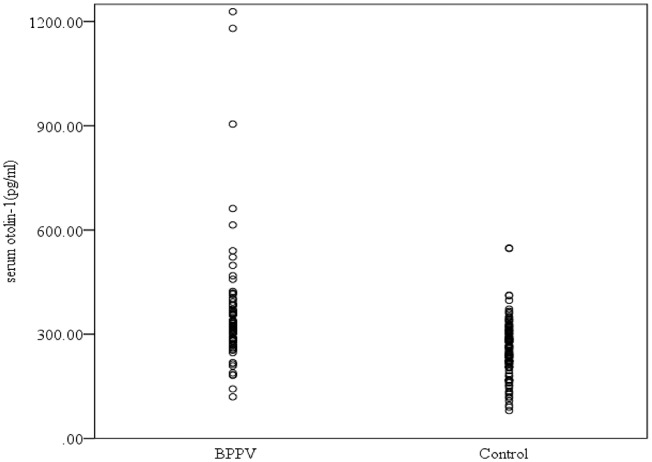
Serum concentrations of otolin-1 in subjects with BPPV and healthy controls.

**Figure 2:**
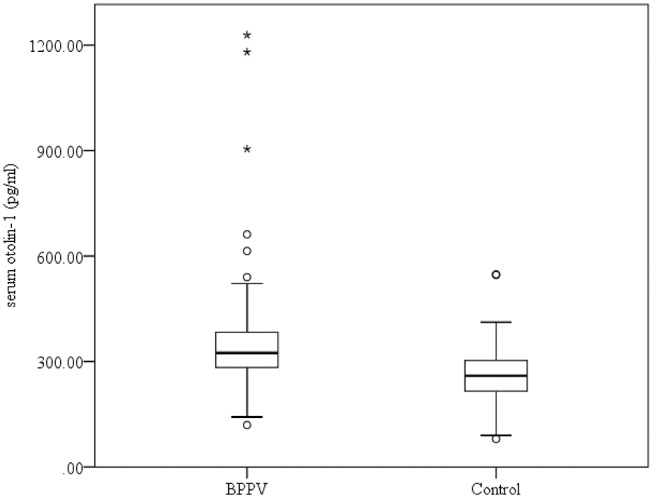
Comparison of serum otolin-1 levels in subjects with BPPV and healthy controls.

**Figure 3:**
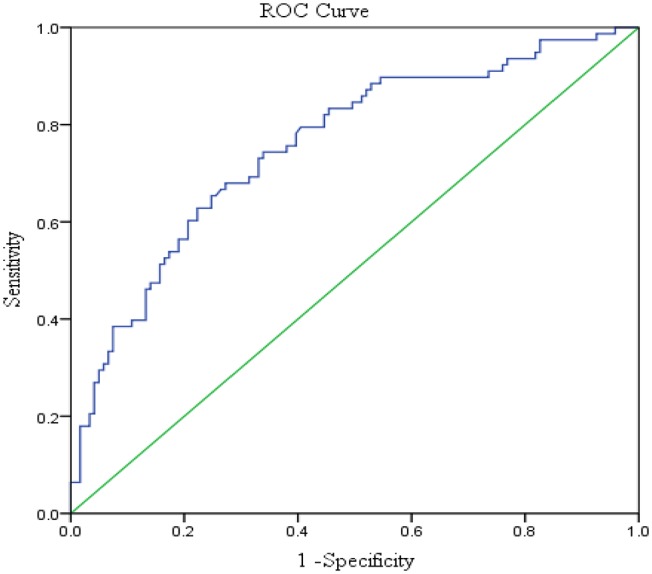
Serum otolin-1 levels provided an area under the curve of 0.757, 95% CI (0.687~0.826) (sensitivity = 67.9%, specificity = 72.7%) for BPPV patients vs. healthy controls.

Serum levels of otolin-1 were significantly higher in BPPV patients [median 324.55 pg/ml (IQR 282.68–383.68)] than in healthy controls [median 259.54 pg/ml (IQR 215.50–305.07)] (*p* < 0.001, Mann–Whitney *U*-test) (Figures 1, 2). To evaluate the potential for serum otolin-1 in the diagnosis of BPPV, ROC analysis was performed. A serum otolin-1 value of 299.45 pg/ml was the optimal cut-off value to discriminate patients with BPPV from healthy controls (area under the curve 0.757, 95% CI 0.687~0.826); this value had a sensitivity of 67.9% and a specificity of 72.7% (Figure 3).

## Discussion

In this pilot study, we measured serum otolin-1 levels in *de novo* patients with idiopathic BPPV and demonstrated that serum otolin-1 levels were significantly higher in these patients than in healthy controls. Furthermore, we found that high levels of serum otolin-1 (>299.45 pg/ml) may serve as a biomarker to differentiate patients with BPPV from control subjects, suggesting the potential use of serum otolin-1 as a biomarker for BPPV episodes.

BPPV, an otoconia-related balance disorder, is the most common cause of vertigo in humans. The diagnosis of BPPV largely relies on a characterized clinical history and positional nystagmus during positive provocative maneuvers. However, this approach may be limited by the availability of trained specialists in view of the high incidence of vertigo. Importantly, in 30% of BPPV cases, management is challenging; this is especially true in patients with subjective BPPV or multiple canal involvement and in patients who find it difficult to perform diagnostic positional maneuvers, such as small children or frail elderly patients. In these cases, expensive and time-consuming investigations are required for inexperienced clinicians to diagnose and treat patients complaining of vertigo or dizziness, and delays in diagnosis or unreasonable treatment may result in increasing costs for patients. In Western countries, medical costs associated with the inappropriate diagnosis and treatment of BPPV can be as high as 2,684.74 US dollars per person (22). In studies from China, the missed or misdiagnosed rate of BPPV was as high as 60%, and the average expenditure was 1232.32 US dollars per patient. It has been estimated that the annual economic burden in Shanghai due to the unreasonable examination and treatment of BPPV was between 198.28 million and 1.14 billion US dollars (23, 24).

It is therefore necessary to explore laboratory markers as tools for achieving the quicker and more accurate diagnosis of BPPV. Various laboratory markers, such as neutrophil-lymphocyte ratios; serum vitamin D, C-reactive protein, DD dimer, fibrinogen, uric acid, and creatine kinase levels; or myocardial type and bone mineral density, have been investigated for the differential diagnosis and prediction of BPPV attacks, but some of these markers show no significant variation and may have no clinical application value (25–31).

Otoliths are dense crystals composed of calcium carbonate and an organic matrix and are primarily involved in gravity sensing by vestibular hair cells. Otoconia are synthesized primarily during embryonic development, and their calcification is essentially completed by the seventh postnatal day. The otoconial complex forms via a dynamic turnover process that occurs throughout an individual's lifetime. Otoconial degeneration and displaced otoconia falling into the canal are the leading causes of BPPV (1, 2, 7). It is known that most episodes of BPPV, even in untreated patients, recover spontaneously within 1–4 weeks because the dislodged otoconia can be dissolved in the endolymph (15, 18, 32). As we were not able to obtain inner ear tissue from the humans in real time, and there is currently no suitable model to study this metabolic process, little is known about it.

Otolin-1 is an inner ear-specific collagen that forms a collagen-like scaffold that promotes optimal otoconia formation. It is a glycoprotein that is specifically secreted by the inner ear, and its messenger mRNA is strictly expressed in the support cells of vestibular maculae, semicircular canal cristae, the organ of Corti, and the marginal cells of the striavascularis (27, 28). Previous studies found that otolin-1 can pass through the labyrinth-blood barrier and enter the peripheral systemic blood circulation. Serum otolin-1 levels significantly increase with age, consistent with the finding that otoconia degeneration was age-related in a mouse model and data showing that the prevalence of BPPV increases with age. A pilot study measured serum otolin-1 levels in patients with BPPV and found that they were significantly higher in affected patients than in healthy controls, with only one-third of patients with BPPV having serum otolin-1 values higher than the control range (15). In addition, that study included only 14 patients with BPPV and 10 healthy controls, and some of the patients were not having an acute episode of vertigo; this may limit the valid generalization of their results. Recently, another clinical study reported that serum otolin-1 levels significantly increased in patients who underwent mastoidectomy due to chronic otitis media and were independently associated with the duration of drilling (33).

In our pilot study, we recruited 78 *de novo* idiopathic patients with BPPV during the attacks of vertigo, and most of these patients were evaluated within a week of symptom onset. We found that otolin-1 levels were significantly higher in the circulation of patients with BPPV than in that of healthy controls. ROC analysis showed that a cut-off value of 299.45 pg/ml of serum otolin-1 had a sensitivity of 67.9% and a specificity of 72.7% for suggestion of a BPPV episode.

These promising results suggested that serum otolin-1 levels may serve as a biomarker for BPPV episodes. However, serum otolin-1 levels cannot suggest which side semicircular canal is affected, and cannot distinguish the status of the displaced otoconia, such as canalithiasis or cupulolithiasis. Therefore, the marker serum otolin-1 has no guiding effect on which type of canalith repositioning maneuver can be used to treat BPPV.

This study, which represents the early phase of biomarker evaluation of BPPV, has some limitations. First, the sample size of the study was relatively small, and most of the study subjects were middle-aged or elderly individuals. No other subjects with vertigo or dizziness were selected as controls. The normal ranges of otolin-1 in healthy subjects and patients with vestibular migraine, vestibular paroxysmia, and neuritis vestibularis are still unknown. Indeed, it is unknown whether an increase in otolin-1 is clinically relevant to vestibular function. Second, the levels of otolin-1 differed among patients with BPPV, and the time between symptom onset and blood sample collection varied. We did not dynamically measure serum otolin-1 levels at different periods in patients with BPPV. It is unclear whether the serum levels of otolin-1 in patients with BPPV are correlated with the recurrence rate because the sample size was too small and the follow-up time was too short to draw specific conclusions. Third, the sensitivity and specificity of otolin-1 are not high. In the future, we will perform follow-ups to determine the otolin-1 levels or other possible biomarkers for otoconia, such as otoconin 90, at different stages of disease with a larger sample bigger study size to overcome the limitations of the current study.

## Conclusion

Serum levels of the otolin-1 protein were significantly higher in patients with BPPV than in healthy controls and may serve as a potential biomarker for BPPV episodes and be used to promote better management of BPPV clinically. However, further studies should be conducted with larger patient cohorts and dynamic assessments of otolin-1 levels in different stages to establish its value.

## Data Availability Statement

The datasets analyzed in this aricle are not publicly available. Requests to access the datasets should be directed to wu_yunqin@126.com.

## Ethics Statement

The studies involving human participants were reviewed and approved by the ethics committee of Hwa Mei Hospital, University of Chinese Academy of Science (protocol number KY-2017-014-03). The patients/participants provided their written informed consent to participate in this study.

## Author Contributions

YW and WH conceived and led the work. WH, WY, XL, MZ, LL, QG, and ZF acquired and checked medical records. YW and WH drafted and revised the manuscript with input from all co-authors.

## Conflict of Interest

The authors declare that the research was conducted in the absence of any commercial or financial relationships that could be construed as a potential conflict of interest.
